# Association Mapping of Main Tomato Fruit Sugars and Organic Acids

**DOI:** 10.3389/fpls.2016.01286

**Published:** 2016-08-26

**Authors:** Jiantao Zhao, Yao Xu, Qin Ding, Xinli Huang, Yating Zhang, Zhirong Zou, Mingjun Li, Lu Cui, Jing Zhang

**Affiliations:** ^1^College of Horticulture, Northwest A&F UniversityYangling, China; ^2^Key Laboratory of Protected Horticultural Engineering in Northwest, Ministry of AgricultureYangling, China; ^3^College of Forestry, Northwest A&F UniversityYangling, China; ^4^College of Food Science and Engineering, Northwest A&F UniversityYangling, China

**Keywords:** tomato, association mapping, sugars, organic acids, metabolites

## Abstract

Association mapping has been widely used to map the significant associated loci responsible for natural variation in complex traits and are valuable for crop improvement. Sugars and organic acids are the most important metabolites in tomato fruits. We used a collection of 174 tomato accessions composed of *Solanum lycopersicum* (123 accessions) and *S. lycopersicum* var *cerasiforme* (51 accessions) to detect significantly associated loci controlling the variation of main sugars and organic acids. The accessions were genotyped with 182 SSRs spreading over the tomato genome. Association mapping was conducted on the main sugars and organic acids detected by gas chromatography-mass spectrometer (GC-MS) over 2 years using the mixed linear model (MLM). We detected a total of 58 significantly associated loci (*P* < 0.001) for the 17 sugars and organic acids, including fructose, glucose, sucrose, citric acid, malic acid. These results not only co-localized with several reported QTLs, including *fru9.1/PV, suc9.1/PV, ca2.1/HS, ca3.1/PV, ca4.1/PV*, and *ca8.1/PV*, but also provided a list of candidate significantly associated loci to be functionally validated. These significantly associated loci could be used for deciphering the genetic architecture of tomato fruit sugars and organic acids and for tomato quality breeding.

## Introduction

Sugars and organic acids are the key components impacting tomato quality and customer preferences. They account for over 60% of the dry matter, and contribute to soluble solid content (SSC) and also are essential to the flavor intensity (Davies et al., 1981; Goff and Klee, 2006; Baldwin et al., 2008; Kader, 2008; Bastias et al., 2011). However, in the long breeding processes, the sugars are usually evaluated by soluble solid content (SSC), and the organic acids are usually evaluated by titratable acid (TA; Saliba-Colombani et al., 2001; Xu et al., 2013; Ruggieri et al., 2014; Sauvage et al., 2014; Zhang et al., 2016). QTL mappings or genome-wide association studies (GWAS) focusing on the individual sugars and organic acids in tomato fruits were quite limited (Fulton et al., 2002; Schauer et al., 2006, 2008; Ruggieri et al., 2014; Sauvage et al., 2014). Besides, in the present available researches focusing on individual sugars and organic acids in tomato fruits, only a few of these metabolites were measured, such as fructose, glucose, sucrose, malic acid, citric acid, ascorbic acid (Osvald et al., 2001; Fulton et al., 2002; Schauer et al., 2005; Ruggieri et al., 2014).

Genome-wide association studies (GWAS) or association mapping have been widely used in identifying candidate QTLs/genes that are related to various agronomically important traits and to uncover the genetic architecture that controls these traits (Atwell et al., 2010; Li et al., 2012; Shirasawa et al., 2013; Chen et al., 2014; Sauvage et al., 2014). The development of metabolomics tools, such as gas chromatography-mass spectrometer (GC-MS) have facilitated the comprehensive phenotyping of complex metabolomic traits (Saito and Matsuda, 2010). Recently the metabolite-based association study has validated the metabolome-GWAS in genetic improvement of complex traits (Riedelsheimer et al., 2012; Chen et al., 2014; Sauvage et al., 2014; Wen et al., 2014; Matsuda et al., 2015). However, the understanding of the genetic and molecular basis of natural variation of tomato fruit sugars and organic is still quite limited (Fulton et al., 2002; Schauer et al., 2008; Sauvage et al., 2014).

Tomato is a major crop plant and a research model system for fruit development and is also an important source of fiber and nutrients in the human diet (Meissner et al., 1997; Giovannoni, 2001; Tomato Genome Consortium, 2012). Many association studies have been published to date for studying the main morphological and nutritional traits in tomato (Mazzucato et al., 2008; Shirasawa et al., 2013; Xu et al., 2013; Ruggieri et al., 2014; Sauvage et al., 2014; Zhang et al., 2015, 2016). However, GWAS or association mapping will probably remain an efficient way of investigating the missing heritability, as the significant associated signals may well define the genomic regions where rare variants, structural variants, and other forms of underlying variation are likely to cluster (Manolio et al., 2009).

In this study, we aimed to investigate the genetic architecture of the main sugars and organic acids in tomato fruits. To reach this objective, fruit sugars and organic acids were evaluated by using GC-MS (Lisec et al., 2006). We then carried out an association mapping study using the mixed linear model (MLM) to detect the significant loci responsible for the natural variations of main sugars and organic acids. We present results on the genotypic diversity, heritability and significantly associated loci of the main sugars and organic acids in tomato.

## Materials and methods

### Plant material

The experiments were performed on 174 tomato accessions comprised of 123 cherry tomato accessions (*Solanum lycopersicum* var. *cerasiforme*) and 51 large-fruit cultivars (*S. lycopersicum*; See Table S1; Zhang et al., 2016). All accessions were grown during the springs of 2013 and 2014, respectively, according to a randomized complete block design with three replicates (10 plants per replicate), as described in Zhang et al. (2016). All accessions received the same horticultural practices. All fruits were harvested at the red-ripe stage each sample consisted of ten fruits for each accession. After quickly removing the seeds, the flash was quickly cut into pieces and were immediately frozen in liquid nitrogen and stored at −80°C until analysis.

### Sugar and organic acid analysis

Extraction and derivatization of sugars, sugar alcohols and organic acids in tomato fruits were mainly according to Zhang et al. (2010) with minor modifications. One hundred milligrams of flash tomato tissue was used in the extraction. Due to the wide range of the concentrations of sugars and organic acids, two vials with different volumes of extract were prepared for each sample, with 5 μL for highly abundant metabolites (fructose, sucrose, glucose, malic acid, etc.) and 100 μL for less abundant metabolites. Briefly, after fractionation of non-polar metabolites into chloroform, 5 and 100 μL of the polar phase of each sample were taken and transferred into separate 2.0 ml Eppendorf vials. These samples were then dried under vacuum without heating and then derivatized with methoxyamine hydrochloride and N-methyl-N-trimethylsilyl-trifluoroacetamide (MSTFA) sequentially (Lisec et al., 2006).

After derivatization, the metabolites were analyzed via an Agilent 7890A GC/5795C MS (Agilent Technology, Palo Alto, CA, USA) with an electron ionization source. One microliter sample was injected and performed at 230°C in splitless mode with helium carrier gas flow at 1 ml/min. Chromatography was performed using a DB-5MS capillary column (20 m × 0.18 mm × 0.18 μm) with a 5 m Duraguard column in front. The temperature program started isothermal at 70°C for 2.471 min and then increased to 330°C by a 10.119°C/min ramp and kept for 2.471 min. Mass spectra were collected at 5.6 scans/s with an m/z 50–600 scanning range. The transfer line temperature and the ion source temperature were set to 250 and 230°C, respectively.

Metabolites were identified by comparing fragmentation patterns with those in a mass spectral library generated on our GC/MS system and an annotated quadrupole GC-MS spectral library from the Golm Metabolome Database (http://csbdb.mpimpgolm.mpg.de/csbdb/gmd/msri/gmd_msri.html). Ribitol was used as the international standard for quantification.

### Association mapping

The DNA of the 174 accessions was extracted from fresh leaf tissue following the method of Fulton et al. (1995). Samples were genotyped with 182 SSR markers (see details in Table S2), as described by Zhang et al. (2016). The protocol for Polymerase Chain Reaction (PCR) and electrophoresis (6% PAGE) was described by Sun et al. (2012). All SSR markers were mainly selected from the SOL Genomics Network (http://sgn.cornell.edu/) and the VegMarks database (http://vegmarks.nivot.affrc.go.jp/). Only markers with minor allele frequency (MAF) > 0.05 were genotyped with the whole accessions (Zhang et al., 2015). Population structure of the 174 tomato accessions was analyzed via STRUCTURE2.3.3 software (Pritchard et al., 2000). We set the number of hypothetical subpopulations (K) at 2–10 in order to evaluate the population structure with an admixture model and the Markov Chain Monte Carlo replicates and the burn-in length was 200,000, 100,000, respectively. We used Evanno transformation method to infer the optimal K of populations (Evanno et al., 2005). The kinship matrix was calculated via SPAGeDi software (Hardy and Vekemans, 2002). We calculated association mapping between markers and phenotypes using the mixed linear model (Q+K model) via TASSEL 2.1 software (Bradbury et al., 2007). Decay of LD and the corresponding significance level (*P*-value) were calculated using TASSEL 2.1 software (Bradbury et al., 2007). We analyzed the metabolic data for GWAS in 2013 and 2014 separately. The raw *P*-values were corrected for multiple tests in order to reduce false positive associations using the Benjamini and Hochberg FDR test (Benjamini and Hochberg, 1995). After *P*-value-correction, we used *P* < 0.005 as the value to detect associations and *P* < 0.001 as the significant value to reduce false positive associations. The amount of phenotypic variation explained by each marker was estimated by *R*^2^.

### Statistics

SAS 8.1 program (SAS institute, Cary, NC) or the R statistical Software (http://www.r-project.org) 3.0.2 were used for statistical analyses. We replaced the values of zero (undetectable) for all metabolites by the smallest non-zero value in the whole dataset (Mathieu et al., 2008; Zhang et al., 2015). All the phenotypes were log_2_-transformed (ng g^−1^ fresh weight h^−1^) before further association mapping analysis. Correlations among sugars and organic acids and other traits were analyzed in the R statistical software and the results were presented via HemI 1.0. We estimated genetic variance, genetic by environment interaction variance, technical variance, and heritability values according to the method of Xu et al. (2013).

## Results

### Phenotyping

In total, 17 sugars, sugar alcohols and organic acids were detected using the 20 μL and 100 μL reaction systems, with eight sugars and nine organic acids, respectively (Table 1). The main sugars in tomato fruits are fructose, glucose, and galactose. The highest concentration was detected on glucose. The concentration of allose and threitol is relatively lower and the lowest concentration was observed on myo-inostiol. Among the eight organic acids, two of them are amino acids (L-proline and L-glutamic acid). Their concentration is relatively lower compared with the other organic acids. The highest concentration was observed on gluconic acid. The concentration of butanedioic acid was also very high. The concentration of citric acid and malic acid were relatively lower, compared with gluconic acid and butanedioic acid. Among all the sugars and organic acids, only some of them were likely to be normally distributed, such as fructose, galactose, myo-inositol, citric acid, gluconic acid, etc. (Figure S1). The heritability of the 17 metabolites varied from 0.293 (hexdecanoic acid) to 0.674 (citric acid). Fructose, glucose, sucrose, citric acid, and malic acid had a higher heritability value compared with the other compounds. So, association mapping was analyzed separately for the metabolite traits in 2013 and 2014.

**Table 1:** **Phenotypic variation of main tomato fruit sugars and organic acids among the 174 tomato accessions**.

**Phenotype**	***H*^2^**	**Max (ppm)**	**Min (ppm)**	**Average (ppm)**	***SD* (ppm)**
Fructose	0.553	77.139	4.878	23.839	12.519
Glucose	0.547	84.682	3.344	16.438	10.398
Sucrose	0.635	5.838	0.053	0.892	1.032
Galactose	0.611	66.073	1.131	13.606	8.409
Myo-inositol	0.382	0.683	0.079	0.268	0.103
Allose	0.468	10.418	1.013	3.806	1.737
Threitol	0.296	41.343	0.054	3.240	4.665
Octanol	0.421	1.398	0.148	0.576	0.287
Citric acid	0.674	10.040	1.527	4.518	1.890
Malic acid	0.668	7.426	0.512	2.106	1.268
L-Proline	0.487	2.621	0.083	0.834	0.523
Butanoic acid	0.531	3.744	0.055	1.002	0.579
L-Glutamic acid	0.429	6.311	0.244	2.185	0.988
Gluconic acid	0.364	33.231	1.495	9.927	4.823
Hexdecanoic acid	0.293	1.890	0.196	0.534	0.228
Octadecanoic acid	0.358	6.770	0.064	1.135	1.007
Butanedioic acid	0.485	16.714	1.192	6.809	3.920

*Maximum (Max), minimum (Min), standard deviation (SD)*.

**Figure 1:**
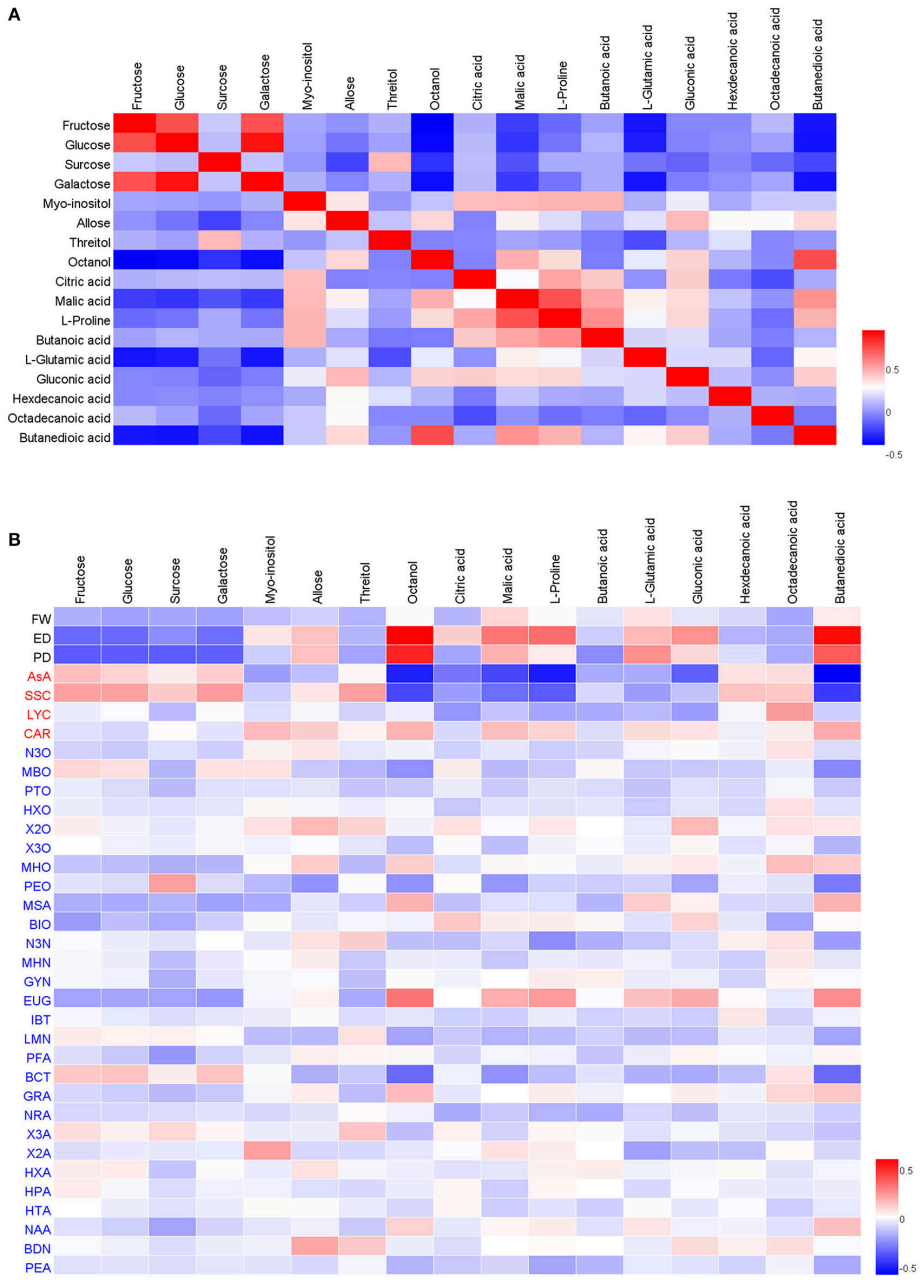
**The Pearson correlation analysis of the main sugars, organic acids, morphological, nutritional, and sensory traits**. Panel **(A)** is the correlation between the main sugars and organics; Panel **(B)** is the correlation between the main sugars, organic acids, and morphological, nutritional and sensory traits. Positive correlations are indicated in red, while negative correlations are indicated in blue.

Pearson correlation coefficients (*r*) among the 17 metabolites revealed that compounds corresponding to a functional classification of the metabolites tended to be positively correlated (Figure 1A). For example, fructose, glucose, and galactose had a significant positive correlation value and were clustered together with each other. The main organic acids, such as citric acid, malic acid, butanoic acid, were also positively clustered together. We observed that main sugars and organic acids were negatively corrected.

### Association mapping

We assessed population structure of the 174 tomato accession using STRUCTURE 2.3.3 software with 182 SSRs. According to Evanno method (Evanno et al., 2005), the 174 tomato accessions could be divided into two sub-populations (Figure S2). The division could be seen as the separation between cherry tomato types and large-fruit types (Zhang et al., 2016). The tomato genome decayed at about 8 cM (Zhang et al., 2015; see Figure S3). Using the mixed linear model in TASSEL under MAF>5%, after Bonferroni correction, 139 marker-trait associations (MTAs) were detected in 2013 (97 MTAs) or 2014 (83 MTAs) and 40 MTAs of them were detected in both years (Table 2, see more details in Table S3). Among these, 58 MTAs had a significant value (*P* < 0.001), with 40, 35 significant associations in 2013, 2014, respectively. We observed at least one MTA on all sugars and organic acids except for threitol. These MTAs were spread unevenly over tomato genome, with all chromosomes carried at least one MTA (chromosome 1, Chr1) and up to 30 MTAs were detected on Chr9. The number of MTAs ranged from zero (no MTAs were observed for threitol) to 38 (citric acid).

**Table 2:** **Association mapping for 17 sugars, sugar alcohols and organic acids in tomato fruit estimated with K+Q (MLM) model on 174 tomato accessions (only those where ***P*** < 0.001 are listed)**.

**Phenotype**	**Locus**	**Chromosome**	**Position^a^**	**2013**		**2014**	
				**Corrected *P*^b^**	***R*^2^**	**Corrected *P***	***R*^2^**
Fructose	TES291	1	62.09	2.44E-04	0.0925	0.003	0.0227
	TES671	5	134.17	ns	–	2.63E-04	0.0501
	TGS2911	6	93.92	0.001	0.0643	4.12E-04	0.0255
	SSR122	6	101	ns	–	4.73E-04	0.037
	TES520	7	0.04	ns	–	9.57E-04	0.0242
	TGS801	9	8.73	ns	–	2.63E-04	0.0801
Glucose	TES671	5	134.17	2.64E-04	0.0893	1.19E-04	0.0725
	TGS2911	6	93.92	5.13E-04	0.0003	5.15E-04	0.0249
	SSR122	6	101.00	2.38E-04	0.0053	1.75E-04	0.0404
	TGS801	9	8.73	0.002	0.0716	5.15E-04	0.0649
Sucrose	TES291	1	62.09	5.76E-04	0.0677	ns	–
	TES835	3	123.55	9.49E-06	0.1694	4.06E-04	0.1212
	TES945	6	87.82	2.80E-04	0.0904	ns	–
	SSR122	6	101.00	0.003	0.0662	5.15E-04	0.0985
	SSR45	7	60.00	2.68E-04	0.0841	6.28E-04	0.0749
	TGS2132	8	19.52	ns	–	6.28E-04	0.0689
	TGS801	9	8.73	8.94E-05	0.1290	2.66E-05	0.1517
	SSR142	9	16.5	ns	–	2.63E-04	0.1274
	SSR110	9	55.7	ns	–	5.15E-04	0.1014
	TES618	12	15.07	2.64E-04	0.0750	0.003	0.0526
Galactose	TES671	5	134.17	8.84E-04	0.0669	4.12E-04	0.0475
	TGS801	9	8.73	ns	–	9.89E-04	0.0529
Allose	TGS821	7	71.48	4.99E-04	0.0551	0.003	0.0293
Octanol	TES291	1	62.09	7.61E-04	0.0284	0.003	0.0269
	SSR133	4	30.6	ns	–	3.69E-12	0.1121
Citric acid	SSR92	1	0	ns	–	2.63E-04	0.0726
	SSR32	2	58.00	2.63E-04	0.0315	0.003	0.0315
	TGS1548	2	77.52	2.30E-04	0.0388	3.74E-04	0.0498
	TES1276	2	82.99	3.07E-04	0.0317	3.78E-04	0.0451
	TGS292	4	65.43	0.001	0.0210	8.98E-04	0.0288
	SSR13	5	28	ns	–	3.74E-04	0.0801
	TGS364	5	46.19	2.73E-04	0.0377	1.19E-04	0.0445
	TGS862	6	32.36	2.51E-04	0.0338	0.002	0.0393
	TES945	6	87.82	0.001	0.0354	2.65E-04	0.0608
	SSR45	7	60.00	8.27E-07	0.0946	4.12E-04	0.0577
	TGS821	7	71.48	2.55E-04	0.0469	5.15E-04	0.0595
	TGS354	8	30.65	2.81E-04	0.0427	0.002	0.042
	TGS607	8	37.89	2.63E-04	0.0362	4.06E-04	0.0581
	TGS947	8	72.56	2.66E-04	0.0433	ns	–
	TES36	9	4.22	2.58E-04	0.0339	1.85E-04	0.0436
	TGS560	9	78.87	2.64E-04	0.0578	ns	–
	TES562	9	92.73	2.64E-04	0.0414	0.003	0.0375
	TGS2885	12	32.04	3.40E-05	0.0007	ns	–
Malic acid	TOM166	9	3.10	7.62E-04	0.0362	ns	–
L-Glutamic acid	TGS827	3	4.42	9.11E-04	0.0458	ns	–
	TES56	3	85.69	8.70E-04	0.0323	ns	–
Gluconic acid	SSR266	1	32.70	2.53E-04	0.1013	ns	–
Octadecanoic acid	TES786	8	99.13	7.42E-04	0.0773	ns	–
Butanedioic acid	TGS207	3	60.74	6.23E-07	0.0904	2.04E-05	0.0564
	SSR43	4	15	ns	–	8.24E-04	0.0124
	TGS821	7	71.48	2.76E-04	0.0254	5.15E-04	0.0169
	SSR344	8	4.00	8.36E-04	0.0437	ns	–
	TOM166	9	3.1	ns	–	9.57E-04	0.0244
	SSR142	9	16.50	7.61E-04	0.0376	ns	–
	SSR110	9	55.70	5.04E-04	0.0344	4.08E-04	0028
	TES623	9	83.56	7.25E-04	0.0223	0.002	0.0143
	TES6	11	49.76	3.83E-04	0.0346	ns	–
	TGS3266	12	50.33	3.67E-04	0.0358	ns	–

a *Genetic distance of the marker was mainly found in EXPEN2000 reference map (http://www.solgenomics.net)*.

b *P-values are corrected following the Benjamini and Hochberg (1995) procedure (see section Materials and Methods)*.

*ns, no significant; –, not given*.

For the eight sugars and sugar alcohols, 56 MTAs were observed with 31, 43 MTAs in 2013 and 2014, respectively. Among these, 18 MTAs were detected in both years. The largest MTA number was detected for sucrose (16 MTAs, either in 2013 or 2014) and no MTA was detected for threitol. For fructose, nine MTAs were observed in total and two of them were observed both in 2013 and 2014. For sucrose, 16 MTAs were observed and the most significant association was detected on TES835 (Chr3) both in 2013 and 2014, explaining 16.94, 12.12% of the phenotypic variation. The other significantly associated marker was TGS801 (Chr9) and was also detected both in 2013 and 2014, explaining 12.9, 15.17% of the phenotypic variation, respectively. For octonol, it was significantly associated with marker SSR133 (Chr 4) in 2014. This association had a very high significance value (*P* = 3.69E-12), accounting for 11.21% of the phenotypic variation. However, no significant association was detected between this marker and octonol variation in 2013.

For the nine organic acids, 83 MTAs were detected, with 66, 50 MTAs in 2013, 2014, respectively. Among these, 33 MTAs were detected in both years. For citric acid, 38 MTAs were detected in 2013 or 2014, representing the largest MTAs for all measured metabolites, with at least one MTA for each chromosome. The most significant association was observed on SSR45 (Chr7) in 2013 (*P* = 8.27E-07), accounting for 9.46% of the phenotypic variation. Besides, this association was also observed in 2014, representing 5.77% of the phenotypic variation. For malic acid, five MTAs were observed either in 2013 or 2014 (Table S3). The most significant association was observed on TOM166 (Chr9) in 2013, accounting for 3.62% of the phenotypic variation. For butanedioic acid, 20 MTAs were detected either in 2013 or 2014, and 10 of them had a significance value (*P* < 0.001). For proline and glutamic acid, the two main amino acids with a high concentration in tomato fruits, four and six MTAs were observed, either in 2013 or 2014, respectively. The two MTAs with a significance value for glutamic acid were TGS827 (Chr3) and TES56 (Chr3) both in 2013, explaining 4.58, 3.23% of the phenotypic variation, respectively. Less MTAs were observed for butanoic acid, gluconic acid, and hexdecanoic acid, with two, one and one MTA, either in 2013 or 2014, respectively.

## Discussion

### Phenotype diversity

In this study, we observed up to 17 sugars, sugar alcohols and organic acids (Table 1), which greatly diversified the reported sugar and organic acid types in tomato fruits. In tomato fruits, there are many different sugars, sugar alcohols, and organic acids and strong Pearson correlation coefficients were observed among these metabolites. This is particularly true for fructose, glucose, and galactose and the main organic acids, such as citric acid, malic acid, and butanoic acid (Figure 1A). Besides, we also observed that the concentration of sugars and organic acids were significantly correlated with some important tomato fruit volatiles, in positive correlations or negative correlations (Figure 1B). Compounds corresponding to the same functional classification of the metabolites tended to be positively correlated, as revealed in our previous studies (Zhang et al., 2015, 2016). These results demonstrate the diverse biological functions of the sugars and organic acids in shaping of tomato fruit quality. The concentrations of fructose, glucose, sucrose, and galactose were negatively correlated with fruit morphological traits, including fruit weight (FW), fruit equatorial diameter (ED), and fruit polar diameter (PD) and were positively correlated with soluble solid content (SSC). Malic acid, citric acid and proline were negatively correlated with ascorbic acid (AsA) and SSC. We also observed some positive or negative correlations between the main sugars and organic acids with the main volatiles. For the complete correlation data, see Table S4. Similar results were also found in other previous studies (Fulton et al., 2002; Ruggieri et al., 2014), demonstrating the complexity of the nutritional characterization and genetic makeup of tomato fruit quality.

### Population structure and LD

Population structure is a strong confounding factor in GWAS and could lead to false positive associations (Pritchard et al., 2000; McCarthy et al., 2008; Nordborg and Weigel, 2008; Shirasawa et al., 2013). Based on 182 SSR markers, the 174 tomato accessions could be divided into two sub-populations and the division could be seen as the separation between cherry tomato types and large-fruit types (Zhang et al., 2016). However, the concentrations of the sugars and organic acids detected in this study varied greatly among the 174 tomato accessions (Table 1). This could be mainly due to the narrow genetic diversity in the tomato breeding history of intensive human selection (Miller and Tanksley, 1990; Lin et al., 2014). In cultivated tomato, LD decays over large genomic regions and could up to several Mb, which is advantageous for GWAS, as fewer markers being able to cover the whole tomato genome (Xu et al., 2013; Sauvage et al., 2014). In this study, the LD decays at about 8 cM and the average marker genome coverage is about 5.2 cM (960/182), demonstrating that using the 182 SSRs is enough to cover the tomato genome. Our previous GWAS confirmed that using these SSRs could detect positive marker-trait associations (Zhang et al., 2015, 2016). Cherry tomato accessions could be seen as a mosaic of wild and cultivated tomato genomes, and could be useful to overcome the high LD for GWAS (Ranc et al., 2012; Xu et al., 2013). Our previous GWAS focused on the main fruit quality traits, such FW, SSC, AsA, LYC, and 28 volatiles in this 174 tomato accessions also confirmed this, demonstrating the feasibility of this study. However, the overall SSRs used in our study is still relatively limited, comparing with the dense SNPs available in tomato genomes. With a higher genome marker density, such as SNPs, GWAS could be used to detect candidate genes directly linked to the metabolic composition of sugars and organic acids in tomato fruits, despite a high LD level and population structure in tomato (Ruggieri et al., 2014; Sauvage et al., 2014; Zhang et al., 2016).

### Association mapping

A total of 56 MTAs were associated with the seven sugars in 2013 (31 MTAs) or 2014 (43 MTAs) with at least two MTAs (myo-inositol) and up to 16 MTAs (sucrose). For the nine organic acids, 83 MTAs were detected in 2013 (66 MTAs) or 2014 (50 MTAs). Fulton et al. (2002) detected 23, 18, and 10 QTLs for fructose, glucose, and sucrose, respectively, using four tomato advanced backcross populations. Beside, they also observed 17 QTLs for citric acid, 20 QTLs for glutamic acid and 21 QTLs for malic acid. Schauer et al. (2008) detected up to 332 QTLs for the main tomato primary metabolites in a tomato IL population, including 104 QTLs for 22 amino acids, 102 QTLs for 22 organic acids, and 39 QTLs for 12 sugars. Among these, fourwere detected for fructose, three for glucose, in all 3 years' field trails, even though only two and one QTLs were detected for citric acid and malic acid, respectively. Our results obtained via a GWAS approach contrasted with these results in terms of the number of QTLs and their chromosome positions. Similar results were also found using a GWAS approach for the 36 metabolite traits in a collection of tomato accessions by Sauvage et al. (2014). Among the 44 significant associations detected within the 36 traits by Sauvage et al. (2014), only two, three significant associations were observed for fructose and sucrose, respectively. For citric acid, malic acid, and proline, only one, two and two significant associations were detected, respectively. This difference could be due to the methodological principles underlying QTL mapping and GWAS and be explained by the more stringent threshold used in GWAS and the confounding effect of population structure (Sauvage et al., 2014). Similar results were also found in Arabidopsis (Chan et al., 2010), maize (Riedelsheimer et al., 2012), and rice (Chen et al., 2013), indicating that GWAS has a larger variability and the linkage mapping relies on a much narrower genetic pool, comparing with association mapping (Riedelsheimer et al., 2012).

**Figure 2:**
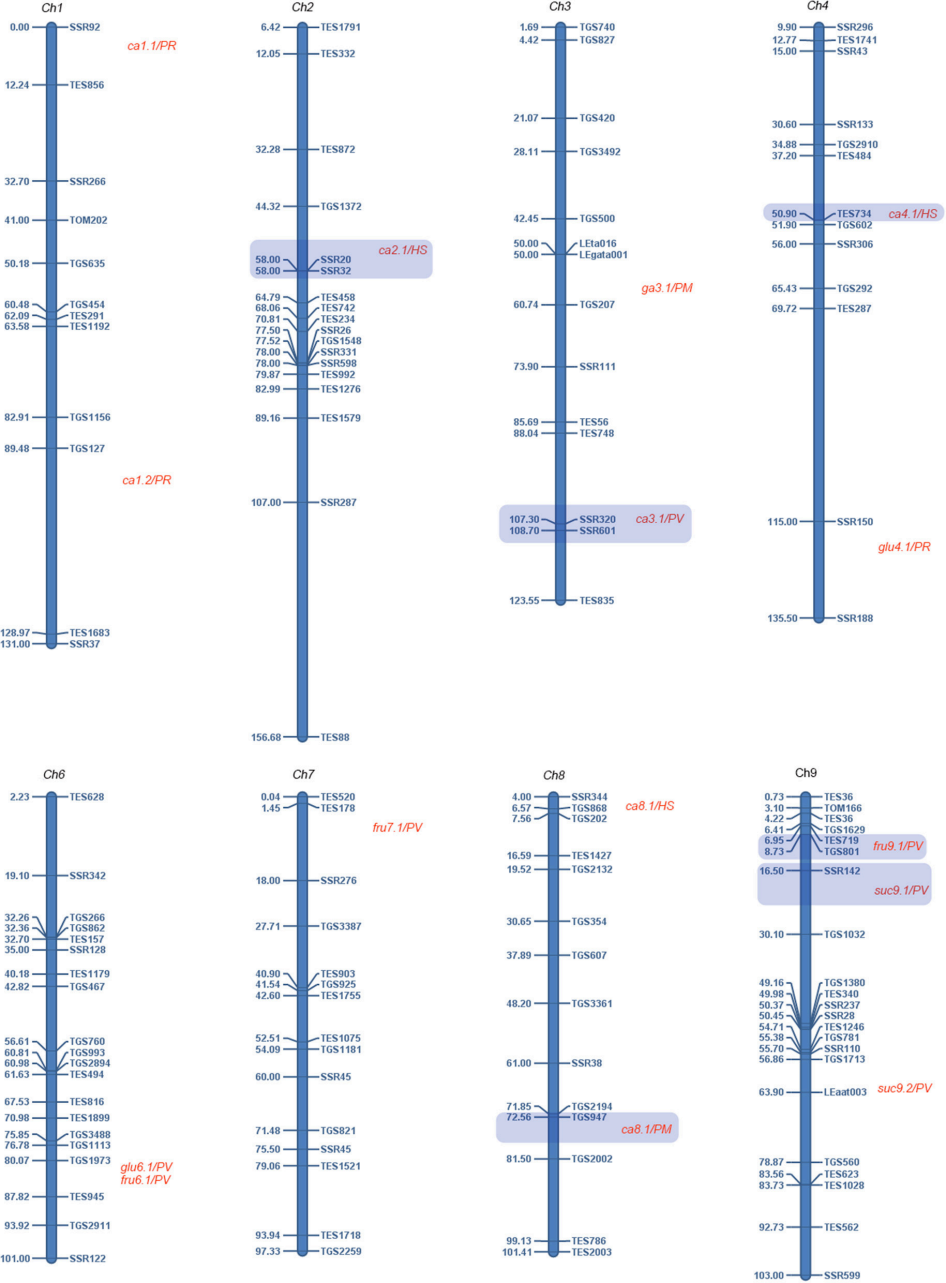
**Comparison of significant associations and co-localized QTLs identified by linkage mapping**. The names and Positions of SSRs are showed on the right and left of the chromosomes, respectively. Co-localized QTLs are mainly reported in Fulton et al. (2002). Fru, fructose; glu, glucose; suc, sucrose; ca, citric acid.

However, our results still confirmed several reported QTLs on main tomato fruit sugars and organic acids (Figure 2). Fructose were associated with two SSRs (TES291 and TGS127) on chromosome one (Chr1). Schauer et al. (2008) detected 39 QTLs for 12 sugars. Among these, one major QTL was detected for fructose on Chr1 in IL1-1-3, across all 3 years' field trails. Fulton et al. (2002) also detected one QTL for fructose on Chr1 at about 131 cM. These results demonstrate that there should be one major QTL for the variation of fructose on Chr1. Besides, there are another three QTLs in Fulton et al. (2002) that are likely to co-localized with the associated loci in this study. For instance, *fru9.1/PV* on Chr9 was located in less than 0.3 cM away from TGS801, and we observed that this loci was significantly associated with the variation of fructose in 2014 and could explain 8.01% of the variation. *fructose6.1/PV* was located about 10 cM away from the significantly associated loci TGS2911. This significantly associated loci only explained 3.7% of the phenotypic variation. This could be mainly due to the large genomic distance between the associated loci and QTL (>8 cM). However, it is still possible that this association might be caused be *fructose6.1/PV*, based on previous GWAS results that tomato genome decays at about 10–20 cM (Mazzucato et al., 2008; van Berloo et al., 2008; Xu et al., 2013; Zhang et al., 2016). Besides, fructose and glucose were both associated with TGS2911, and Fulton et al. (2002) reported there was also a QTL (*glu6.1/PV*) for glucose near TGS2911. This observation suggests that in the near region of TGS2911, there are either two dependent QTLs/genes or one gene involved in the sugar metabolic pathways. Sucrose was significantly associated with four loci on chr9 (TGS801, SSR142, SSR110, and TES1028). Fulton et al. (2002) reported two QTLs (*suc9.1/PV* and *suc9.2/PV*) for sucrose on Chr9. *suc9.1/PV* was located about 5 cM away from the significantly associated loci SSR142. *suc9.2/PV* was located about 7 cM away from the significantly associated loci SSR110. These two significant associations accounted for 12.74, 10.14% of the variation of sucrose in 2014 and could be mainly due to *suc9.1/PV* and *suc9.2/PV*, respectively.

Citric acid was associated with up to 38 loci with at least one MTA on each chromosome, representing the largest number of MTAs for all traits. Among these, 18 MTAs had a significance value (*P* < 0.001). Fulton et al. (2002) detected up to 17 QTLs for citric acid. Among these, we confirmed at least four of them, including *ca2.1/HS, ca3.1/PV, ca4.1/HS*, and *ca8.1/PM*. The significantly associated loci SSR32 was about 3 cM away from *ca2.1/HS*, accounting for 3.15% of the phenotypic variation. The significantly associated loci SSR601 was about 1 cM away from *ca3.1/PV* and TES734 was less than 1 cM away from *ca4.1/HS*. TGS947 was about 6 cM away from *ca8.1/PM*. Since the LD of the tomato population decayed at about 8 cM based on 182 SSRs (Zhang et al., 2015), these three significant associations could be mainly caused by *ca2.1/HS, ca3.1/PV*, and *ca8.1/PM*. Besides, we also detected three MTAs on chr1 (SSR92, TGS1156, and TGS127). *ca1.1/PR* and *ca1.2/PR* are two QTLs on Chr1 detected in Fulton et al. (2002) in the near region of SSR92 and TGS127 (<8 cM). This suggested that the two significantly associated loci SSR92 and TGS127 could also be caused by *ca1.1/PR* and *ca1.2/PR*.

Many primary and secondary metabolites including sugars and organic acids in tomato fruits have a relatively low heritability (Schauer et al., 2008; Sauvage et al., 2014; Zhang et al., 2015, 2016). However, even those traits exhibiting a low heritability could still be valuable targets for fruit quality breeding purposes (Schauer et al., 2008). Apart from fructose, glucose, sucrose, malic acid, citric acid, attentions should also be paid to the other sugars and organic acids, such as galactose, butanoic acid, and butanedioic acid, etc. GWAS will probably remain an efficient way of investigating the remaining heritability. Because the available associations may well define the genomic regions of rare variants, structural variants and other forms of underlying variation (Manolio et al., 2009).

## Conclusion

The association mapping approach undertaken allowed the detection of 58 significant associations for the main tomato fruit sugars and organic acids. These metabolites are essential for deciphering the genetic architecture of tomato fruit nutritional composition. Our findings suggested that using SSRs and the mixed linear model (MLM) were suitable for detecting significant associations with tomato fruit sugars and organic acids. Several formerly identified QTLs, such as *fru9.1/PV, suc9.1/PV, ca2.1/HS, ca3.1/PV, ca4.1/HS*, and *ca8.1/PM* were co-localized with a group of significant associated loci, which validated this study. Most of the sugars and organic had a relatively low heritability. Further GWAS will probably remain an efficient way in investigating the remaining heritability and detecting more significantly associated loci for tomato fruit sugars and organic acids.

## Author contributions

JTZ, JZ, and ZZ designed the study. JTZ and YX carried out the main GC-MS analysis and molecular mapping, analyzed the data, and drafted the manuscript. XH, QD, ML, LC participate in the data analysis of sugars and organic acids. YZ participated in genotyping. All authors corrected and approved the final version.

## Funding

This work was supported by the Program for New Century Excellent Talents in University (No. NCET-12-0474), National Natural Science Foundation of China (Grant No. 31301498), Agricultural Science and Technology Innovation and Research in Shaanxi province (Grant No. 2016NY-165) and the National Agricultural Science Foundation (No. 201203002).

### Conflict of interest statement

The authors declare that the research was conducted in the absence of any commercial or financial relationships that could be construed as a potential conflict of interest.

## Supplementary material

Table S1 **The number and origin of the whole accessions**.

Table S2 **Detailed information for 182 SSR markers used for association mapping in this research**.

Table S3 **Genome-wide associations for 17 sugars, sugar alcohols and organic acids in tomato fruit estimated with K+Q (MLM) model on 174 tomato accessions (only those where ***P*** < 0.005 are listed)**.

Table S4 **The Pearson correlation coefficients (***r***) among sugars, organic acids, nutritional traits, and volatiles**.

Figure S1 **Frequency distribution of the content of the detected 17 sugars and organic acids**.

Figure S2 **Optimal K of the population structure of all accessions based on 182 SSR markers, according to Evanno method (2005)**.

Figure S3 **Estimates of LD (***r***^**2**^) over genetic distance on all chromosomes for all 174 tomato accessions**.
