# Supplementary material for: Association Mapping of Main Tomato Fruit Sugars and Organic Acids
Source: Front Plant Sci. 2016 Aug 26;7:1286. doi: 10.3389/fpls.2016.01286 (PMC4999453; doi:10.3389/fpls.2016.01286)
Supplement: Table S1 — The number and origin of the whole accessions. [file Table1.DOCX]

***Supplementary Material***

**Genome-wide association of main tomato fruit sugars and organic acids**

**Jiantao Zhao^1,2†^, Yao Xu^3†^, Qin Ding^1^, Xinli Huang^1,2^, Yating Zhang^1,2^, Zhirong Zou^1,2^, Mingjun Li^1^, Lu Cui^4^, Jing Zhang^1^***

^1^College of Horticulture, Northwest A&F University, Yangling, Shaanxi, China

^2^Key Laboratory of Protected Horticultural Engineering in Northwest, Ministry of Agriculture, Yangling, Shaanxi, China

^3^College of Forestry, Northwest A&F University, Yangling, Shaanxi, China

^4^College of Food Science and Engineering, Northwest A&F University, Yangling, Shaanxi, China

**^†^** These two authors contribute equally to the present study

*** Correspondence:** Jing Zhang, Key Laboratory of Protected Horticultural Engineering in Northwest, College of Horticulture, Northwest A&F University, No. 3 Taicheng Road, Yangling, Shaanxi, 712100, China.

yyzhj@nwsuaf.edu.cn

**Table S1** The number and origin of the whole accessions

| Accession code | Accession name | Origin | Species |
| --- | --- | --- | --- |
| B1 | MTF-1 | Henan, China | *S. lycopersicum var cerasiforme* |
| B2 | MTF-2 | Henan, China | *S. lycopersicum var cerasiforme* |
| B3 | MTF-3 | Henan, China | *S. lycopersicum var cerasiforme* |
| B4 | MTF-4 | Henan, China | *S. lycopersicum var cerasiforme* |
| B5 | MTF-5 | Henan, China | *S. lycopersicum var cerasiforme* |
| B6 | MTF-6 | Henan, China | *S. lycopersicum var cerasiforme* |
| B7 | MTF-7 | Henan, China | *S. lycopersicum var cerasiforme* |
| B8 | MTF-8 | Henan, China | *S. lycopersicum var cerasiforme* |
| B9 | MTF-9 | Henan, China | *S. lycopersicum var cerasiforme* |
| B10 | MTF-10 | Henan, China | *S. lycopersicum var cerasiforme* |
| B11 | MTF-11 | Henan, China | *S. lycopersicum var cerasiforme* |
| B12 | MTF-12 | Henan, China | *S. lycopersicum var cerasiforme* |
| B13 | MTF-13 | Henan, China | *S. lycopersicum var cerasiforme* |
| B14 | MTF-14 | Henan, China | *S. lycopersicum var cerasiforme* |
| B15 | MTF-15 | Henan, China | *S. lycopersicum var cerasiforme* |
| B16 | MTF-16 | Henan, China | *S. lycopersicum var cerasiforme* |
| B17 | MTF-17 | Henan, China | *S. lycopersicum var cerasiforme* |
| B18 | MTF-18 | Henan, China | *S. lycopersicum var cerasiforme* |
| B19 | MTF-19 | Henan, China | *S. lycopersicum var cerasiforme* |
| B20 | MTF-20 | Henan, China | *S. lycopersicum var cerasiforme* |
| B21 | MTF-21 | Henan, China | *S. lycopersicum var cerasiforme* |
| B22 | MTF-22 | Henan, China | *S. lycopersicum var cerasiforme* |
| B23 | MTF-23 | Henan, China | *S. lycopersicum var cerasiforme* |
| B24 | MTF-24 | Henan, China | *S. lycopersicum var cerasiforme* |
| B25 | MTF-25 | Henan, China | *S. lycopersicum var cerasiforme* |
| B26 | MTF-26 | Henan, China | *S. lycopersicum var cerasiforme* |
| B27 | MTF-27 | Henan, China | *S. lycopersicum var cerasiforme* |
| B28 | MTF-28 | Henan, China | *S. lycopersicum var cerasiforme* |
| B29 | MTF-29 | Hebei,China | *S. lycopersicum var cerasiforme* |
| B30 | MTF-30 | Hebei,China | *S. lycopersicum var cerasiforme* |
| B31 | MTF-31 | Hebei,China | *S. lycopersicum var cerasiforme* |
| B32 | MTF-32 | Hebei,China | *S. lycopersicum var cerasiforme* |
| B33 | MTF-33 | Hebei,China | *S. lycopersicum var cerasiforme* |
| B34 | MTF-34 | Hebei,China | *S. lycopersicum var cerasiforme* |
| B35 | MTF-35 | Hebei,China | *S. lycopersicum var cerasiforme* |
| B36 | MTF-36 | Hebei,China | *S. lycopersicum var cerasiforme* |
| B37 | MTF-37 | Hebei,China | *S. lycopersicum var cerasiforme* |
| B38 | MTF-38 | Hebei,China | *S. lycopersicum var cerasiforme* |
| B39 | MTF-39 | Hebei,China | *S. lycopersicum var cerasiforme* |
| B40 | MTF-40 | Hebei,China | *S. lycopersicum var cerasiforme* |
| B41 | MTF-41 | Hebei,China | *S. lycopersicum var cerasiforme* |
| B42 | MTF-42 | Hebei,China | *S. lycopersicum var cerasiforme* |
| B43 | MTF-43 | Hebei,China | *S. lycopersicum var cerasiforme* |
| B44 | MTF-44 | Hebei,China | *S. lycopersicum var cerasiforme* |
| B45 | MTF-45 | Hebei,China | *S. lycopersicum var cerasiforme* |
| B46 | MTF-46 | Hebei,China | *S. lycopersicum var cerasiforme* |
| B47 | MTF-47 | Hebei,China | *S. lycopersicum var cerasiforme* |
| B48 | MTF-48 | Hebei,China | *S. lycopersicum var cerasiforme* |
| B49 | MTF-49 | Hebei,China | *S. lycopersicum var cerasiforme* |
| B50 | MTF-50 | Hebei,China | *S. lycopersicum var cerasiforme* |
| B51 | MTF-51 | Hebei,China | *S. lycopersicum var cerasiforme* |
| B52 | MTF-52 | Hebei,China | *S. lycopersicum var cerasiforme* |
| B53 | MTF-53 | Hebei,China | *S. lycopersicum var cerasiforme* |
| B54 | MTF-54 | Jinnan,China | *S. lycopersicum var cerasiforme* |
| B55 | MTF-55 | Jinnan,China | *S. lycopersicum var cerasiforme* |
| B56 | MTF-56 | Jinnan,China | *S. lycopersicum var cerasiforme* |
| B57 | MTF-57 | Jinnan,China | *S. lycopersicum var cerasiforme* |
| B58 | MTF-58 | Jinnan,China | *S. lycopersicum var cerasiforme* |
| B59 | MTF-59 | Jinnan,China | *S. lycopersicum var cerasiforme* |
| B60 | MTF-60 | Jinnan,China | *S. lycopersicum var cerasiforme* |
| B61 | MTF-61 | Jinnan,China | *S. lycopersicum var cerasiforme* |
| B62 | MTF-62 | Jinnan,China | *S. lycopersicum var cerasiforme* |
| B63 | MTF-63 | Jinnan,China | *S. lycopersicum var cerasiforme* |
| B64 | MTF-64 | Jinnan,China | *S. lycopersicum var cerasiforme* |
| B65 | MTF-65 | Beijing,China | *S. lycopersicum var cerasiforme* |
| B66 | MTF-66 | Beijing,China | *S. lycopersicum var cerasiforme* |
| B67 | MTF-67 | Beijing,China | *S. lycopersicum var cerasiforme* |
| B68 | MTF-68 | Beijing,China | *S. lycopersicum var cerasiforme* |
| B69 | MTF-69 | Beijing,China | *S. lycopersicum var cerasiforme* |
| B70 | MTF-70 | Beijing,China | *S. lycopersicum var cerasiforme* |
| B71 | MTF-71 | Beijing,China | *S. lycopersicum var cerasiforme* |
| B72 | MTF-72 | Beijing,China | *S. lycopersicum var cerasiforme* |
| B73 | MTF-73 | Beijing,China | *S. lycopersicum var cerasiforme* |
| B74 | MTF-74 | Beijing,China | *S. lycopersicum var cerasiforme* |
| B75 | MTF-75 | Beijing,China | *S. lycopersicum var cerasiforme* |
| B76 | MTF-76 | Beijing,China | *S. lycopersicum var cerasiforme* |
| B77 | MTF-88 | Bejing,China | *S. lycopersicum var cerasiforme* |
| B78 | MTF-89 | Bejing,China | *S. lycopersicum var cerasiforme* |
| B79 | MTF-90 | Bejing,China | *S. lycopersicum var cerasiforme* |
| B80 | MTF-91 | Bejing,China | *S. lycopersicum var cerasiforme* |
| B81 | MTF-92 | Bejing,China | *S. lycopersicum var cerasiforme* |
| B82 | LS-127 | Bejing,China | *S. lycopersicum var cerasiforme* |
| B83 | LS-137 | Bejing,China | *S. lycopersicum* |
| B84 | LS-78 | Bejing,China | *S. lycopersicum var cerasiforme* |
| B85 | LS-77 | Bejing,China | *S. lycopersicum var cerasiforme* |
| B86 | LS-84 | Bejing,China | *S. lycopersicum* |
| B87 | LS-79 | Bejing,China | *S. lycopersicum var cerasiforme* |
| B88 | MTF-93 | Bejing,China | *S. lycopersicum var cerasiforme* |
| B89 | MTF-77 | Xinjiang,China | *S. lycopersicum var cerasiforme* |
| B90 | MTF-78 | Xinjiang,China | *S. lycopersicum var cerasiforme* |
| B91 | MTF-79 | Xinjiang,China | *S. lycopersicum var cerasiforme* |
| B92 | MTF-80 | Xinjiang,China | *S. lycopersicum var cerasiforme* |
| B93 | MTF-81 | Xinjiang,China | *S. lycopersicum var cerasiforme* |
| B94 | MTF-82 | Xinjiang,China | *S. lycopersicum var cerasiforme* |
| B95 | MTF-83 | Xinjiang,China | *S. lycopersicum var cerasiforme* |
| B96 | MTF-84 | Xinjiang,China | *S. lycopersicum var cerasiforme* |
| B97 | MTF-85 | Xinjiang,China | *S. lycopersicum var cerasiforme* |
| B98 | MTF-86 | Xinjiang,China | *S. lycopersicum var cerasiforme* |
| B99 | MTF-87 | Xinjiang,China | *S. lycopersicum var cerasiforme* |
| B100 | LS-1 | Shanghai,China | *S. lycopersicum var cerasiforme* |
| B101 | LS-2 | Shanghai,China | *S. lycopersicum var cerasiforme* |
| B102 | TTI2603A | Shanghai,China | *S. lycopersicum var cerasiforme* |
| B103 | LS-4 | Shanghai,China | *S. lycopersicum var cerasiforme* |
| B104 | LS-5 | Shanghai,China | *S. lycopersicum var cerasiforme* |
| B105 | LS-6 | Shanghai,China | *S. lycopersicum var cerasiforme* |
| B106 | LS-9 | Guangzhou,China | *S. lycopersicum* |
| B107 | LS-135 | Guangzhou,China | *S. lycopersicum var cerasiforme* |
| B108 | LS-125 | Guangzhou,China | *S. lycopersicum var cerasiforme* |
| B109 | LS-131 | Guangzhou,China | *S. lycopersicum var cerasiforme* |
| B110 | LS-132 | Guangzhou,China | *S. lycopersicum var cerasiforme* |
| B111 | LS-10 | Guangzhou,China | *S. lycopersicum* |
| B112 | LS-51 | Guangzhou,China | *S. lycopersicum* |
| B113 | LS-7 | Guangzhou,China | *S. lycopersicum* |
| B114 | LS-42 | Guangzhou,China | *S. lycopersicum* |
| B115 | LS-49 | Guangzhou,China | *S. lycopersicum* |
| B116 | LS-148 | Guangzhou,China | *S. lycopersicum* |
| B117 | LS-139 | Guangzhou,China | *S. lycopersicum* |
| B118 | LS-13 | Guangzhou,China | *S. lycopersicum* |
| B119 | LS-33 | Guangzhou,China | *S. lycopersicum* |
| B120 | LS-46 | Guangzhou,China | *S. lycopersicum* |
| B121 | LS-53 | Guangzhou,China | *S. lycopersicum* |
| B122 | LS-43 | Guangzhou,China | *S. lycopersicum* |
| B123 | LS-54 | Guangzhou,China | *S. lycopersicum* |
| B124 | LS-8 | Guangzhou,China | *S. lycopersicum* |
| B125 | LS-126 | Guangzhou,China | *S. lycopersicum var cerasiforme* |
| B126 | LS-128 | GEMANY | *S. lycopersicum var cerasiforme* |
| B127 | LS-136 | GEMANY | *S. lycopersicum* |
| B128 | LS-133 | GEMANY | *S. lycopersicum* |
| B129 | LS-87 | GEMANY | *S. lycopersicum* |
| B130 | LS-24 | GEMANY | *S. lycopersicum var cerasiforme* |
| B131 | LS-11 | GEMANY | *S. lycopersicum* |
| B132 | LS-161 | GEMANY | *S. lycopersicum var cerasiforme* |
| B133 | LS-94 | ISRAEL | *S. lycopersicum var cerasiforme* |
| B134 | LS-92 | ISRAEL | *S. lycopersicum* |
| B135 | LS-93 | ISRAEL | *S. lycopersicum* |
| B136 | LS-86 | ISRAEL | *S. lycopersicum* |
| B137 | LS-88 | ISRAEL | *S. lycopersicum* |
| B138 | LS-90 | ISRAEL | *S. lycopersicum* |
| B139 | LS-87 | ISRAEL | *S. lycopersicum* |
| B140 | LS-91 | ISRAEL | *S. lycopersicum* |
| B141 | LS-89 | ISRAEL | *S. lycopersicum* |
| B142 | LS-85 | ISRAEL | *S. lycopersicum* |
| B143 | LS-56 | ISRAEL | *S. lycopersicum* |
| B144 | LS-150 | ISRAEL | *S. lycopersicum* |
| B145 | LS-58 | ISRAEL | *S. lycopersicum* |
| B146 | LS-65 | ISRAEL | *S. lycopersicum var cerasiforme* |
| B147 | LS-57 | ISRAEL | *S. lycopersicum* |
| B148 | LS-83 | Shanxi,China | *S. lycopersicum var cerasiforme* |
| B149 | LS-67 | Shanxi,China | *S. lycopersicum var cerasiforme* |
| B150 | LS-149 | Shanxi,China | *S. lycopersicum* |
| B151 | LS-45 | Shanxi,China | *S. lycopersicum* |
| B152 | LS-152 | Shanxi,China | *S. lycopersicum* |
| B153 | LS-70 | Shanxi,China | *S. lycopersicum* |
| B154 | LS-25 | Shanxi,China | *S. lycopersicum var cerasiforme* |
| B155 | LS-68 | Shanxi,China | *S. lycopersicum var cerasiforme* |
| B156 | LS-63 | Shanxi,China | *S. lycopersicum var cerasiforme* |
| B157 | LS-55 | Shanxi,China | *S. lycopersicum* |
| B158 | LS-122 | Shanxi,China | *S. lycopersicum var cerasiforme* |
| B159 | LS-64 | USA | *S. lycopersicum var cerasiforme* |
| B160 | LS-41 | USA | *S. lycopersicum* |
| B161 | LS-145 | USA | *S. lycopersicum* |
| B162 | LS-50 | USA | *S. lycopersicum* |
| B163 | LS-30 | USA | *S. lycopersicum* |
| B164 | LS-12 | USA | *S. lycopersicum* |
| B165 | LS-81 | USA | *S. lycopersicum var cerasiforme* |
| B166 | LS-69 | FRANCE | *S. lycopersicum* |
| B167 | LS-151 | FRANCE | *S. lycopersicum* |
| B168 | LS-138 | FRANCE | *S. lycopersicum* |
| B169 | LS-66 | FRANCE | *S. lycopersicum var cerasiforme* |
| B170 | LS-35 | FRANCE | *S. lycopersicum* |
| B171 | LS-40 | FRANCE | *S. lycopersicum* |
| B172 | LS-38 | FRANCE | *S. lycopersicum* |
| B173 | LS-146 | FRANCE | *S. lycopersicum* |
| B174 | LS-44 | FRANCE | *S. lycopersicum* |
