# Supplementary material for: Association Mapping of Main Tomato Fruit Sugars and Organic Acids
Source: Front Plant Sci. 2016 Aug 26;7:1286. doi: 10.3389/fpls.2016.01286 (PMC4999453; doi:10.3389/fpls.2016.01286)
Supplement: Table S3 — Genome-wide associations for 17 sugars, sugar alcohols and organic acids in tomato fruit estimated with K+Q (MLM) model on 174 tomato accessions (only those where P < 0.005 are listed). [file Table3.DOCX]

***Supplementary Material***

**Genome-wide association of main tomato fruit sugars and organic acids**

**Jiantao Zhao^1,2†^, Yao Xu^3†^, Qin Ding^1^, Xinli Huang^1,2^, Yating Zhang^1,2^, Zhirong Zou^1,2^, Mingjun Li^1^, Lu Cui^4^, Jing Zhang^1^***

^1^College of Horticulture, Northwest A&F University, Yangling, Shaanxi, China

^2^Key Laboratory of Protected Horticultural Engineering in Northwest, Ministry of Agriculture, Yangling, Shaanxi, China

^3^College of Forestry, Northwest A&F University, Yangling, Shaanxi, China

^4^College of Food Science and Engineering, Northwest A&F University, Yangling, Shaanxi, China

**^†^** These two authors contribute equally to the present study

*** Correspondence:** Jing Zhang, Key Laboratory of Protected Horticultural Engineering in Northwest, College of Horticulture, Northwest A&F University, No. 3 Taicheng Road, Yangling, Shaanxi, 712100, China.

yyzhj@nwsuaf.edu.cn

Table S3 Genome-wide associations for 17 sugars, sugar alcohols and organic acids in tomato fruit estimated with K+Q (MLM) model on 174 tomato accessions (only those where *P*<0.005 are listed).

| Phenotype | Locus | Chromosome | Position^a^ | 2013 | | 2014 | |
| --- | --- | --- | --- | --- | --- | --- | --- |
|  |  |  |  | Corrected *P*^b^ | R^2^ | Corrected *P* | R^2^ |
| Fructose | TES291 | 1 | 62.09 | 2.44E-04 | 0.0925 | 0.003 | 0.0227 |
|  | TGS127 | 1 | 89.48 | ns | - | 0.003 | 0.0692 |
|  | TES671 | 5 | 134.17 | ns | - | 2.63E-04 | 0.0501 |
|  | TGS2911 | 6 | 93.92 | 0.001 | 0.0643 | 4.12E-04 | 0.0255 |
|  | SSR122 | 6 | 101 | ns | - | 4.73E-04 | 0.037 |
|  | TES520 | 7 | 0.04 | ns | - | 9.57E-04 | 0.0242 |
|  | TGS801 | 9 | 8.73 | ns | - | 2.63E-04 | 0.0801 |
|  | TES623 | 9 | 83.56 | ns | - | 0.004 | 0.0484 |
|  | TES562 | 9 | 92.73 | ns | - | 0.003 | 0.0311 |
| Glucose | TES671 | 5 | 134.17 | 2.64E-04 | 0.0893 | 1.19E-04 | 0.0725 |
|  | TES816 | 6 | 67.53 | 0.004 | 0.0300 | 0.002 | 0.0323 |
|  | TGS2911 | 6 | 93.92 | 5.13E-04 | 0.0003 | 5.15E-04 | 0.0249 |
|  | SSR122 | 6 | 101.00 | 2.38E-04 | 0.0053 | 1.75E-04 | 0.0404 |
|  | TES520 | 7 | 0.04 | 0.002 | 0.0520 | 0.003 | 0.02 |
|  | TGS1181 | 7 | 54.09 | ns | - | 0.002 | 0.0542 |
|  | TGS947 | 8 | 72.56 | 0.004 | 0.0456 | 0.003 | 0.0277 |
|  | TGS801 | 9 | 8.73 | 0.002 | 0.0716 | 5.15E-04 | 0.0649 |
|  | TES562 | 9 | 92.73 | ns | - | 0.003 | 0.0396 |
| Sucrose | TES291 | 1 | 62.09 | 5.76E-04 | 0.0677 | ns | - |
|  | TES835 | 3 | 123.55 | 9.49E-06 | 0.1694 | 4.06E-04 | 0.1212 |
|  | TES945 | 6 | 87.82 | 2.80E-04 | 0.0904 | ns | - |
|  | TGS2911 | 6 | 93.92 | ns | - | 0.003 | 0.0543 |
|  | SSR122 | 6 | 101.00 | 0.003 | 0.0662 | 5.15E-04 | 0.0985 |
|  | SSR276 | 7 | 18.00 | ns | - | 0.002 | 0.087 |
|  | SSR45 | 7 | 60.00 | 2.68E-04 | 0.0841 | 6.28E-04 | 0.0749 |
|  | TGS821 | 7 | 71.48 | ns | - | 0.004 | 0.0475 |
|  | TES1427 | 8 | 16.59 | 0.004 | 0.0411 | ns | - |
|  | TGS2132 | 8 | 19.52 | ns | - | 6.28E-04 | 0.0689 |
|  | TGS801 | 9 | 8.73 | 8.94E-05 | 0.1290 | 2.66E-05 | 0.1517 |
|  | SSR142 | 9 | 16.5 | ns | - | 2.63E-04 | 0.1274 |
|  | SSR110 | 9 | 55.7 | ns | - | 5.15E-04 | 0.1014 |
|  | TES1028 | 9 | 83.73 | 0.002 | 0.0546 | ns | - |
|  | TES618 | 12 | 15.07 | 2.64E-04 | 0.0750 | 0.003 | 0.0526 |
|  | TGS838 | 12 | 92.90 | 0.001 | 0.0799 | ns | - |
| Galactose | SSR150 | 4 | 115.00 | 0.003 | 0.0360 | ns | - |
|  | TES671 | 5 | 134.17 | 8.84E-04 | 0.0669 | 4.12E-04 | 0.0475 |
|  | TGS2911 | 6 | 93.92 | ns | - | 0.002 | 0.0247 |
|  | SSR122 | 6 | 101 | ns | - | 0.002 | 0.0406 |
|  | TGS801 | 9 | 8.73 | ns | - | 9.89E-04 | 0.0529 |
|  | TES562 | 9 | 92.73 | 0.001 | 0.0592 | ns | - |
| Myo-inositol | TGS1548 | 2 | 77.52 | ns | - | 0.003 | 0.0274 |
|  | TES1276 | 2 | 82.99 | ns | - | 0.003 | 0.0267 |
| Allose | TGS821 | 7 | 71.48 | 4.99E-04 | 0.0551 | 0.003 | 0.0293 |
|  | SSR344 | 8 | 4 | ns | - | 0.004 | 0.0284 |
|  | TGS2132 | 8 | 19.52 | ns | - | 0.004 | 0.0216 |
|  | TGS801 | 9 | 8.73 | ns | - | 0.003 | 0.0182 |
|  | TES623 | 9 | 83.56 | 0.004 | 0.0321 | 0.003 | 0.0276 |
| Octanol | TES291 | 1 | 62.09 | 7.61E-04 | 0.0284 | 0.003 | 0.0269 |
|  | SSR598 | 2 | 78.00 | 0.002 | 0.0268 | ns | - |
|  | SSR133 | 4 | 30.6 | ns | - | 3.69E-12 | 0.1121 |
|  | SSR323 | 5 | 12.3 | ns | - | 0.001 | 0.0249 |
|  | TGS821 | 7 | 71.48 | 0.002 | 0.0211 | ns | - |
|  | TGS2132 | 8 | 19.52 | 0.004 | 0.0186 | ns | - |
|  | SSR110 | 9 | 55.70 | 0.002 | 0.0310 | ns | - |
|  | TES623 | 9 | 83.56 | 0.001 | 0.0241 | ns | - |
|  | TES562 | 9 | 92.73 | 0.003 | 0.0208 | ns | - |
| Citrate | SSR92 | 1 | 0 | ns | - | 2.63E-04 | 0.0726 |
|  | TGS1156 | 1 | 82.91 | 0.003 | 0.0298 | ns | - |
|  | TGS127 | 1 | 89.48 | ns | - | 0.002 | 0.049 |
|  | TES872 | 2 | 32.28 | 0.004 | 0.0160 | ns | - |
|  | SSR32 | 2 | 58.00 | 2.63E-04 | 0.0315 | 0.003 | 0.0315 |
|  | TGS1548 | 2 | 77.52 | 2.30E-04 | 0.0388 | 3.74E-04 | 0.0498 |
|  | TES992 | 2 | 79.87 | 0.004 | 0.0143 | ns | - |
|  | TES1276 | 2 | 82.99 | 3.07E-04 | 0.0317 | 3.78E-04 | 0.0451 |
|  | TGS740 | 3 | 1.69 | 0.001 | 0.0230 | 0.003 | 0.0339 |
|  | SSR601 | 3 | 108.70 | 0.002 | 0.0170 | ns | - |
|  | TES484 | 4 | 37.20 | 0.002 | 0.0161 | ns | - |
|  | TES734 | 4 | 50.90 | 0.002 | 0.0161 | ns | - |
|  | TGS292 | 4 | 65.43 | 0.001 | 0.0210 | 8.98E-04 | 0.0288 |
|  | TES287 | 4 | 69.72 | 0.003 | 0.0149 | ns | - |
|  | SSR13 | 5 | 28 | ns | - | 3.74E-04 | 0.0801 |
|  | TGS364 | 5 | 46.19 | 2.73E-04 | 0.0377 | 1.19E-04 | 0.0445 |
|  | TGS645 | 5 | 60.23 | 0.004 | 0.0146 | ns | - |
|  | TES358 | 5 | 60.96 | 0.001 | 0.0215 | ns | - |
|  | TGS862 | 6 | 32.36 | 2.51E-04 | 0.0338 | 0.002 | 0.0393 |
|  | TES157 | 6 | 32.70 | 0.001 | 0.0210 | ns | - |
|  | TGS467 | 6 | 42.82 | 0.001 | 0.0252 | 0.002 | 0.0727 |
|  | TGS1973 | 6 | 80.07 | 0.004 | 0.0289 | ns | - |
|  | TES945 | 6 | 87.82 | 0.001 | 0.0354 | 2.65E-04 | 0.0608 |
|  | SSR45 | 7 | 60.00 | 8.27E-07 | 0.0946 | 4.12E-04 | 0.0577 |
|  | TGS821 | 7 | 71.48 | 2.55E-04 | 0.0469 | 5.15E-04 | 0.0595 |
|  | TES1427 | 8 | 16.59 | 0.003 | 0.0255 | ns | - |
|  | TGS354 | 8 | 30.65 | 2.81E-04 | 0.0427 | 0.002 | 0.042 |
|  | TGS607 | 8 | 37.89 | 2.63E-04 | 0.0362 | 4.06E-04 | 0.0581 |
|  | TGS947 | 8 | 72.56 | 2.66E-04 | 0.0433 | ns | - |
|  | TES36 | 9 | 4.22 | 2.58E-04 | 0.0339 | 1.85E-04 | 0.0436 |
|  | TGS1629 | 9 | 6.41 | 0.001 | 0.0267 | ns | - |
|  | TES719 | 9 | 6.95 | ns | - | 0.004 | 0.0333 |
|  | TGS801 | 9 | 8.73 | 0.001 | 0.0247 | 0.003 | 0.0352 |
|  | TGS560 | 9 | 78.87 | 2.64E-04 | 0.0578 | ns | - |
|  | TES562 | 9 | 92.73 | 2.64E-04 | 0.0414 | 0.003 | 0.0375 |
|  | TES1407 | 10 | 42.91 | ns | - | 0.003 | 0.039 |
|  | SSR80 | 11 | 16.80 | 0.004 | 0.0249 | ns | - |
|  | TGS2885 | 12 | 32.04 | 3.40E-05 | 0.0007 | ns | - |
| Malate | TGS207 | 3 | 60.74 | 0.001 | 0.0365 | ns | - |
|  | TES62 | 5 | 73.73 | ns | - | 0.004 | 0.0285 |
|  | SSR342 | 6 | 19.1 | ns | - | 0.002 | 0.0422 |
|  | TOM166 | 9 | 3.10 | 7.62E-04 | 0.0362 | ns | - |
|  | TGS3266 | 12 | 50.33 | 0.001 | 0.0265 | ns | - |
| L-Proline | TES332 | 2 | 12.05 | 0.002 | 0.0082 | ns | - |
|  | TES945 | 6 | 87.82 | ns | - | 0.002 | 0.0243 |
|  | TGS821 | 7 | 71.48 | 0.004 | 0.0103 | ns | - |
|  | TGS947 | 8 | 72.56 | 0.002 | 0.0123 | ns | - |
| Butanoic acid | SSR342 | 6 | 19.1 | ns | - | 0.002 | 0.0627 |
|  | TGS947 | 8 | 72.56 | 0.002 | 0.0249 | ns | - |
| L-Glutamic acid | TGS827 | 3 | 4.42 | 9.11E-04 | 0.0458 | ns | - |
|  | TGS207 | 3 | 60.74 | 0.004 | 0.0824 | ns | - |
|  | TES56 | 3 | 85.69 | 8.70E-04 | 0.0323 | ns | - |
|  | TGS1606 | 5 | 119.37 | ns | - | 0.002 | 5.95E-06 |
|  | TGS1181 | 7 | 54.09 | ns | - | 0.002 | 0.0301 |
|  | TGS879 | 12 | 31.82 | ns | - | 0.004 | 8.80E-06 |
| Gluconic acid | SSR266 | 1 | 32.70 | 2.53E-04 | 0.1013 | ns | - |
| Hexdecanoic acid | LEta016 | 3 | NG^c^ | 0.0034 | 0.1076 | ns | - |
| Octadecanoic acid | SSR266 | 1 | 32.70 | 0.003 | 0.0662 | ns | - |
|  | SSR342 | 6 | 19.10 | 0.002 | 0.0791 | ns | - |
|  | TGS1113 | 6 | 76.78 | 0.001 | 0.0646 | 0.003 | 0.0536 |
|  | TES786 | 8 | 99.13 | 7.42E-04 | 0.0773 | ns | - |
|  | TGS801 | 9 | 8.73 | 0.004 | 0.0560 | ns | - |
|  | SSR74 | 11 | 40.00 | 0.004 | 0.0660 | ns | - |
| Butanedioic acid | TGS207 | 3 | 60.74 | 6.23E-07 | 0.0904 | 2.04E-05 | 0.0564 |
|  | SSR43 | 4 | 15 | ns | - | 8.24E-04 | 0.0124 |
|  | TES1741 | 4 | 12.77 | ns | - | 0.004 | 0.0076 |
|  | SSR133 | 4 | 30.60 | 0.003 | 0.0343 | ns | - |
|  | SSR323 | 5 | 12.30 | 0.001 | 0.0298 | ns | - |
|  | SSR41 | 5 | 34 | ns | - | 0.004 | 0.0196 |
|  | SSR128 | 6 | 35.00 | 0.004 | 0.0340 | ns | - |
|  | TGS821 | 7 | 71.48 | 2.76E-04 | 0.0254 | 5.15E-04 | 0.0169 |
|  | SSR45 | 7 | 75.50 | 0.003 | 0.0259 | ns | - |
|  | SSR344 | 8 | 4.00 | 8.36E-04 | 0.0437 | ns | - |
|  | TGS2132 | 8 | 19.52 | 0.002 | 0.0180 | 0.004 | 0.0116 |
|  | TES786 | 8 | 99.13 | 0.002 | 0.0263 | ns | - |
|  | TOM166 | 9 | 3.1 | ns | - | 9.57E-04 | 0.0244 |
|  | TGS801 | 9 | 8.73 | ns | - | 0.004 | 0.0078 |
|  | SSR142 | 9 | 16.50 | 7.61E-04 | 0.0376 | ns | - |
|  | SSR110 | 9 | 55.70 | 5.04E-04 | 0.0344 | 4.08E-04 | 0028 |
|  | TES623 | 9 | 83.56 | 7.25E-04 | 0.0223 | 0.002 | 0.0143 |
|  | TES562 | 9 | 92.73 | 0.001 | 0.0204 | 0.003 | 0.0133 |
|  | TES6 | 11 | 49.76 | 3.83E-04 | 0.0346 | ns | - |
|  | TGS3266 | 12 | 50.33 | 3.67E-04 | 0.0358 | ns | - |
| ^a^Genetic distance of the marker was not found in EXPEN2000 reference map (http://www.solgenomics.net).  *^b^P* values are corrected following the Benjamini & Hochberg (2000) procedure (see Materials and Methods).  ^c^Genetic distance of the marker was not found in EXPEN2000 reference map (http://www.solgenomics.net).  ns, no significant; - not given | | | | | | | |
