# Supplementary figures and images for: Association Mapping of Main Tomato Fruit Sugars and Organic Acids
Source: Front Plant Sci. 2016 Aug 26;7:1286. doi: 10.3389/fpls.2016.01286 (PMC4999453; doi:10.3389/fpls.2016.01286)

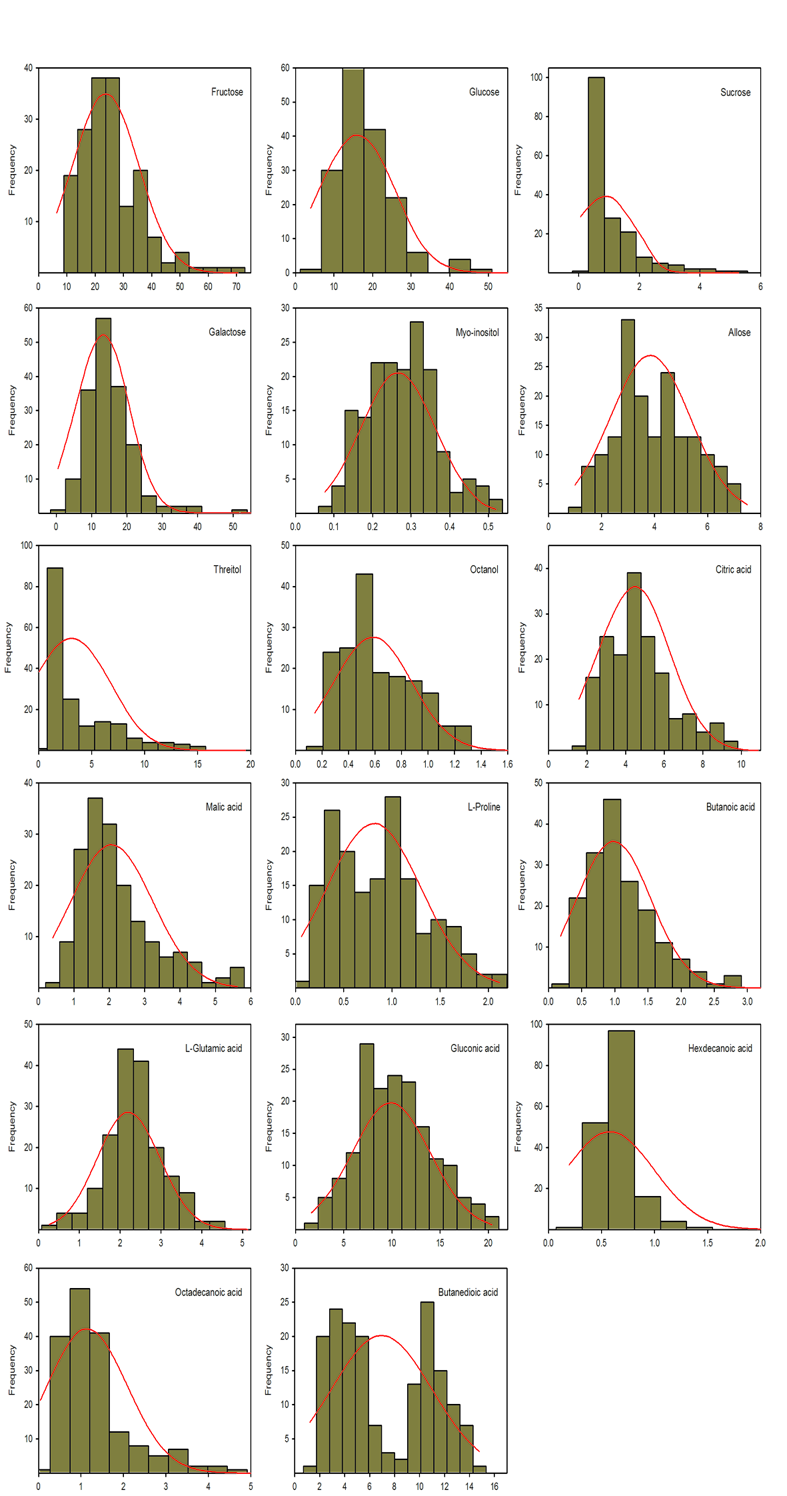

Supplement: Figure S1 — Frequency distribution of the content of the detected 17 sugars and organic acids. [file Image1.TIF]

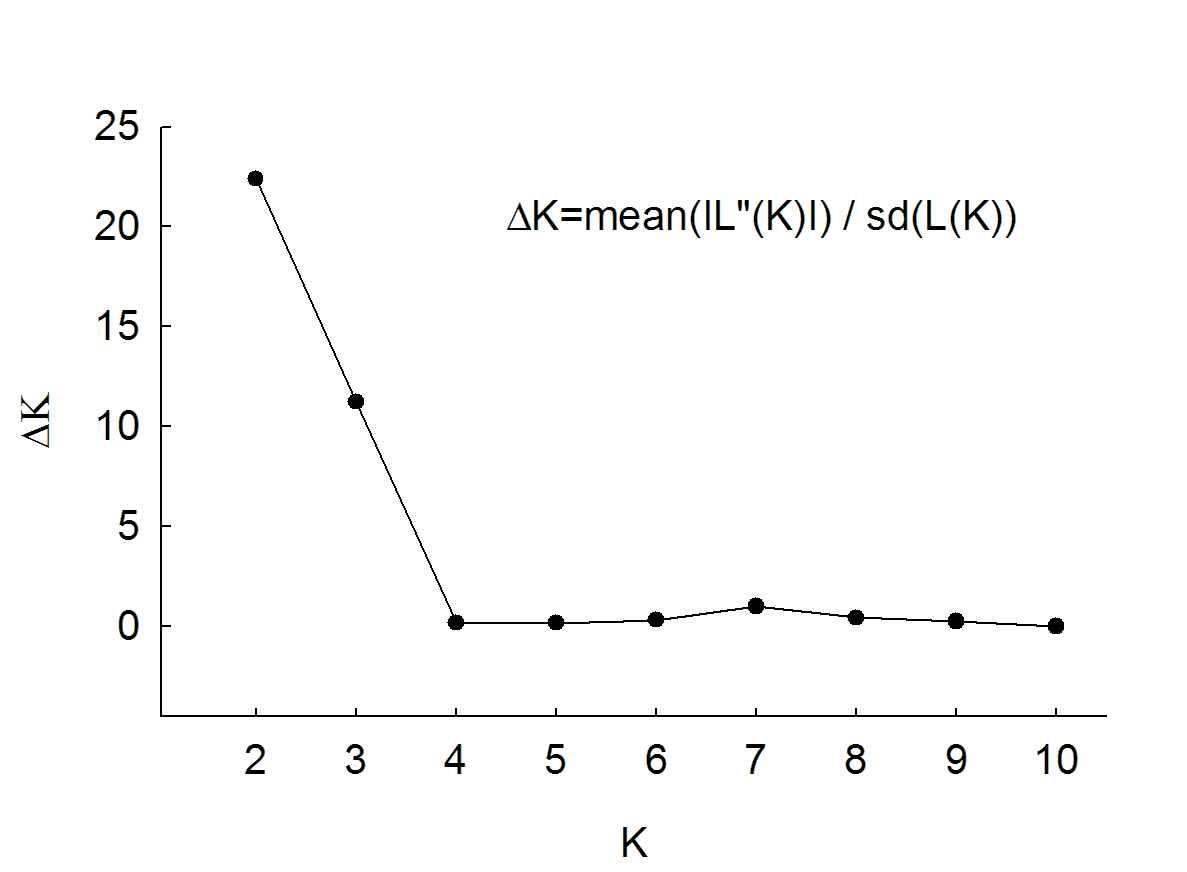

Supplement: Figure S2 — Optimal K of the population structure of all accessions based on 182 SSR markers, according to Evanno method (2005). [file Image2.TIF]

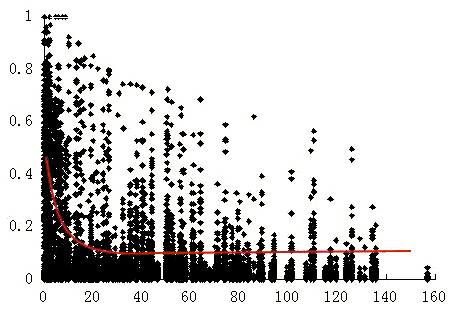

Supplement: Figure S3 — Estimates of LD (r2) over genetic distance on all chromosomes for all 174 tomato accessions. [file Image3.jpg]
